# Human Development XIII: The Connection Between the Structure of the Overtone System and the Tone Language of Music. Some Implications for Our Understanding of the Human Brain

**DOI:** 10.1100/tsw.2008.8

**Published:** 2008-07-13

**Authors:** Søren Ventegodt, Tyge Dahl Hermansen, Isack Kandel, Joav Merrick

**Affiliations:** ^1^ Quality of Life Research Center, Classensgade 11C, 1 sal, DK-2100 Copenhagen O, Denmark; ^2^ Research Clinic for Holistic Medicine, Copenhagen, Denmark; ^3^ Nordic School of Holistic Medicine, Copenhagen, Denmark; ^4^ Scandinavian Foundation for Holistic Medicine, Sandvika, Norway; ^5^ Interuniversity College, Graz, Austria; ^6^ Faculty of Social Sciences, Department of Behavioral Sciences, Ariel University Center of Samaria, Ariel, Israel; ^7^ National Institute of Child Health and Human Development, Jerusalem, Israel; ^8^ Office of the Medical Director, Division for Mental Retardation, Ministry of Social Affairs, Jerusalem, Israel; ^9^ Kentucky Children's Hospital, University of Kentucky, Lexington, USA

**Keywords:** human development, quality of life, QOL, holistic biology, theoretical biology, music theory

## Abstract

The functioning brain behaves like one highly-structured, coherent, informational field. It can be popularly described as a “coherent ball of energy”, making the idea of a local highly-structured quantum field that carries the consciousness very appealing. If that is so, the structure of the experience of music might be a quite unique window into a hidden quantum reality of the brain, and even of life itself. The structure of music is then a mirror of a much more complex, but similar, structure of the energetic field of the working brain. This paper discusses how the perception of music is organized in the human brain with respect to the known tone scales of major and minor. The patterns used by the brain seem to be similar to the overtones of vibrating matter, giving a positive experience of harmonies in major. However, we also like the minor scale, which can explain brain patterns as fractal-like, giving a symmetric “downward reflection” of the major scale into the minor scale. We analyze the implication of beautiful and ugly tones and harmonies for the model. We conclude that when it comes to simple perception of harmonies, the most simple is the most beautiful and the most complex is the most ugly, but in music, even the most disharmonic harmony can be beautiful, if experienced as a part of a dynamic release of musical tension. This can be taken as a general metaphor of painful, yet meaningful, and developing experiences in human life.

## INTRODUCTION

Music, a complex cultural phenomenon of sounds, tones, harmonies, and rhythm, must be understood by the way it is created, and the way it influences the human being listening to it, in the context it is used. Many other vertebrates, however, besides the human being (e.g., songbirds and whales), use a language of tones and rhythms when they communicate. Therefore, in this paper, we will describe music as a phenomenon of nature, closely linked to biology and, of course, especially the nervous system of vertebrates.

As music has a highly mathematic structure, we suggest that it mirrors a mathematical functional aspect of the vertebrate brain. Most interestingly, music seems to have a simple, mathematical fundament independent of the vertebrate morphology and brain size, which might give us a clue to understanding the functioning brain and, more specifically, a new way to understand the universal language of rhythms and tones. In this paper, we will investigate the elements of mathematics and morphology that create the foundation of music.

Music can be analyzed into its elements: rhythm, tone, and sound; harmony, melody, and harmonic tension; and words, meaning, and intended effect. In this paper, we will primarily discuss the principles behind the formation of tones and harmonies, but it is very important for the understanding of music as a whole that we also discuss the principles behind rhythm and sound, because these, like the language of tones, are also important and products of nature. Therefore, to understand some of the basic problems concerning music, we begin with investigating what sound and rhythm really are.

### What is Sound?

Sound is formed by swinging bodies' rhythmic movements of the air. Such movements influence the liquids of the inner ear to move, caused by vibrations in the eardrum spread through the hammer, anvil, and stirrup[1]. This movement makes a "permanent" wave in the cochlea[1]. Membrane basilaris is affected and swings around its normal main position by which hair cells are activated and send off impulses to the ganglion tympani[1]. From there, the signals are sent to the cortex cerebri for processing and interpretation[1]. Simultaneously, the cortex cerebri sends signals to the ganglion tympani – that functions as its own "tiny brain" of the ear – that, via these signals, modifies the activity of the brain in a way that is highly complicated and not fully understood[2].

Thus, we cannot look at the ear as an object of measurement that is objective, but instead as an instrument with a high degree of selectivity, that only registers impulses of interest for the brain. However, it is also possible that the ear's neural system, to a certain degree, "by itself" is able to produce the specific signal needed by the brain, but to find out about this, more experimental research is needed. This means that a fundamental trait of sound is an interpretation of external signals reaching the ear through the molecules of the air.

### What is Rhythm?

Noise is a sound that cannot be organized by the brain. The rhythmic organization of the noise is a splitting up into beats. The beat is a pulse of sound separated by a silence, noise, or sound of different intensity or quality. Rhythm is an organization of the time by a series of beats, partitioning the time into intervals[3]. If we accelerate beat, we will get a transition from rhythm to tone. This transition happens at 10 Hz. Formerly, it was perceived that this transition happened at 20 Hz[4], but our own experiences in the laboratory indicates 10 Hz as the transition border.

A very interesting phenomenon that can be observed by this is transition between quantity and quality. This transition illustrates how our brain interprets reality; where quantities grow in intensity and finally get incomprehensibly large, we find the interface between the consciousness and the subconsciousness. In this way, the phenomena will not appear any more available for the analytical mind, but still available as experiential phenomena through the senses.

## What is a Tone?

A body that vibrates between 10 and 20,000 Hz makes movements in the air that form the tones. The body vibrates sinusoidally, i.e. in the simplest manner something is able to vibrate in. Such tone is ideally seen as absolutely pure because it is not contaminated by other tones vibrating simultaneously with it[5].

### *Overtones and the Vibrating String*

Fig. 1 shows a vibrating string, just as that used by the ancient Greeks as a starting point for understanding the tonal side of music. The ideal vibration, the sinus curve, forms when the string vibrates between maximum and minimum. However, bodies that vibrate act more advanced because it is not only the end of the string that stands more or less still when it vibrates. Here and there, on the nodes along the string, the string stands more still than it does in other places along the string. In Figs. 1–3, only the activity around the nodes is shown.

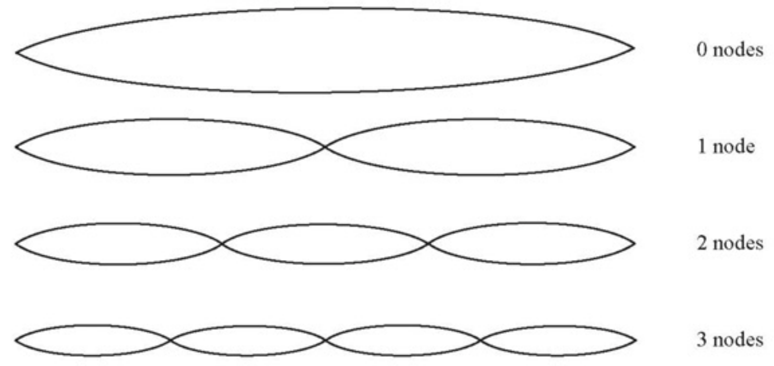

**FIGURE 1**. A swinging string's node points. This system is developed so the swings correspond to 1, 2, 3, etc. nodes. Each node has its own swing speed, corresponding to its frequencies, equivalent to the original string's frequency multiplied by the amount of nodes. By this, a number of tones, called the number of overtones, are formed (see also Fig. 2).

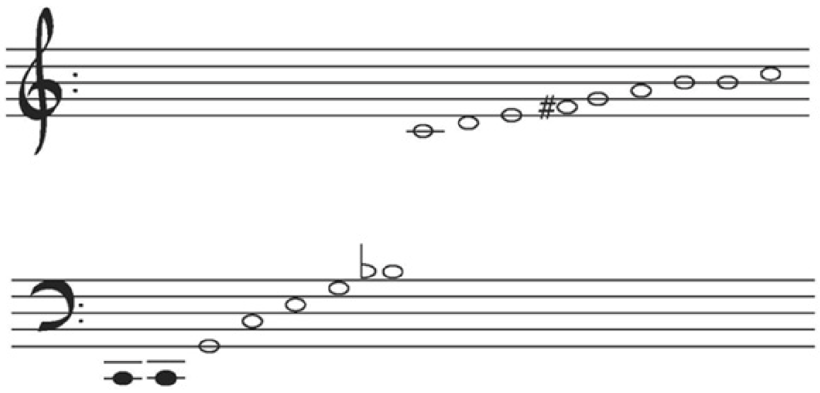

**FIGURE 2.** The number of overtones (nodes give only an approximate description).

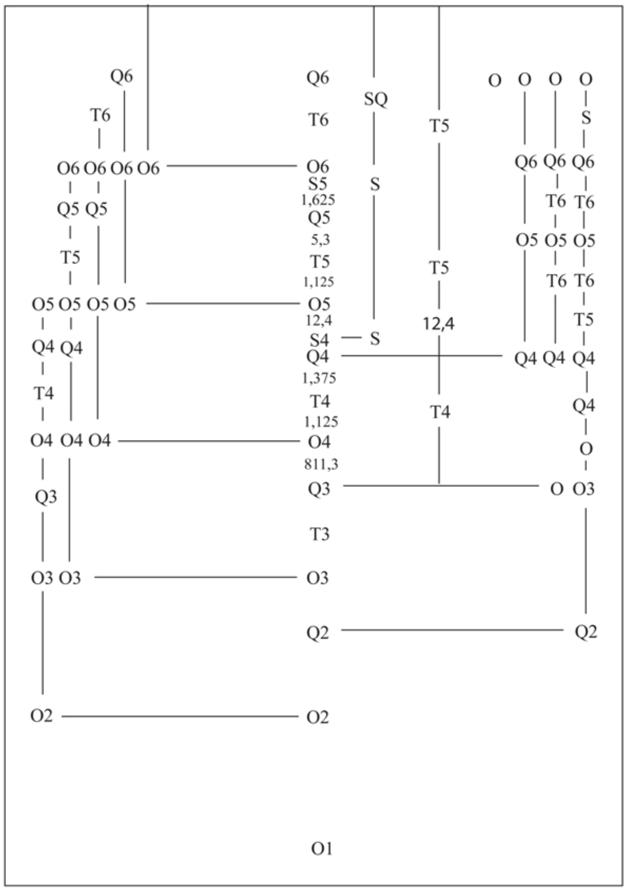

**FIGURE 3**. The tree of overtones. The image is shown as its pattern would look on a millimeter paper.

## What are the Qualities of Sound?

To understand a component that makes sound, we most understand (1) the attack and the specific form through which the sound is created, and (2) the quality of the created sound.

Sound is created by the specific form of the instrument and reflects the materials of the instrument, which have their own specific spectrum of overtones. In theory, all overtones are always present, but each swinging body strengthens and individually hampers the spectrum of overtones. This gives every individual instrument its own sound – just think of the Stradivarius violin. The sound is closely related to the overtones from the materials, and both sound and tone depend on the particular instrument's form[6].

When a string swings, different tones can be distinguished. Almost all can hear the clean fifth that appears with the swing created by two nodes and a portioning in three parts of the string. Many people can also hear the huge clean third, although it demands some exercise to separate the highest overtones from the sound. Therefore, the sound is particularly formed by the seventh and higher overtones (from the seventh or higher nodes) (Professor Yngve Trade, personal communication). Seven nodes are equivalent to the node B (approximately 30 cents too low, see below), with the C as the ground tone.

Through the second transition from quantity to quality, tone is transferred to sound. This time, multiple tones are transferred and interpreted as sound because the brain does not have the capacity to interpret too many tones otherwise. Because it is limited in how much complexity the consciousness is able to deal with simultaneously, this phenomenon can be defined as tolerance broadness. The brain may distinguish between rhythm, tone, and sound[7].

## The Relationship Between Wavelength, Frequency, and the Cent Value of the Sound

A specific length of a string captures a specific swing speed (corresponding to a specific wavelength). This swing speed is dependent on the string material, thickness, restringing, temperature, and other physical relationships. The wavelength is reversed proportionally with the frequency. This means that if the wavelength is halved, then the frequency is doubled, and vice versa. The closest overtone is found by doubling the frequency, corresponding to an octave and sensed as the keynote. This makes a sound, sounding as if the difference represented the distance between the octaves. Seemingly, the tone system is grounded on such repetition because the keynote and the octave are considered as the same tone[5].

A frequency of octaves divided into 12 equal pieces of 100 cents gives the equally floating temperate chromatic scale[8,9]. Such division of octaves is necessary to compare the heights of two tones because both the wavelengths and the frequencies are misleading. This is the case because the same difference in frequency or wavelength makes a different tone distance if the tones are located differently in the system of octaves. The ear is built to grasp the height of the tone as a logarithmic function of the frequency. This means that when the frequency is doubled multiple times, this is only experienced as a doubling of the tone height.

## The Intervals and the Extended System of Overtones

The system of overtones is more complicated than illustrated in Fig. 2 because every overtone in an ideally elastic material in itself is the keynote of its own overtones[5]. In this way, a complete, highly-branched tree of overtones is formed like a fractal system, where a specific pattern is repeated in itself on all levels (see Fig. 3).

This simple tree can be represented in a simpler way by focusing on the fifth and third, and their overtones (see Fig. 4). This figure shows all tones involved in a specific tone height and compared to the keynote (each line represents a triad). The figure is illustrated through the clean fifths and thirds, where the most intervals (the distance between the horizontal lines) are formed around quarter tones and, therefore, do not form a nice tonal system.

Concerning the trimmed truncated overtone trees, we search for a scale (as we know from the C major scale) or intervals enough to form a scale. The truncated tree of overtones can be adapted to the system (see Fig. 5). By this adaptation, the row in which fifths and thirds go up has no connotation with the tone height reached. By this, the fifths third is the same tone as the thirds fifth, and the third's third fifth equal with the fifth's third third or the third's fifth third. Because we work in clean fifths and thirds, we will never find the same tone more than once through the system that includes 1 + 2 + 3 + 4 + … + 13 (= 82) tones totally.

Such a system is extremely complex because all included tones are different. However, it is possible to make such a system simpler if the lowest corner is drawn by T(12) (as if the system was a plate of rubber), so the T(3), T(6), T(9), and T(12) are going to be the octaves above the keynote, and Q(12), at the same time, is pressed with the top side a bit up. In this way also, Q(12) forms an octave and a system with only 12 tones is created. This illustrates our 12-tone system. By this, the third and fifth get a size called temperate in the equally floating temperate scale – the same as Johann Sebastian Bach (1685–1750) used because of its huge degree of freedom. This freedom is exactly illustrated by the freedom to move around in the system as illustrated in Fig. 5.

If all the fifths and thirds are now plotted onto a millimeter paper (illustrated by the drawing in Fig. 4), a simple system compared to the natural overtone system is formed (see Fig. 6).

To define the tones for an ordinary C major is difficult work because, for example, the tone F is defined as QQQTT, a very complicated composition. However, we do not have direct admission to other intervals than fifths and (huge) thirds under such circumstances, and it is only these and the octave that are conceived as harmony between the overtones (caused by the truncation of the sound).

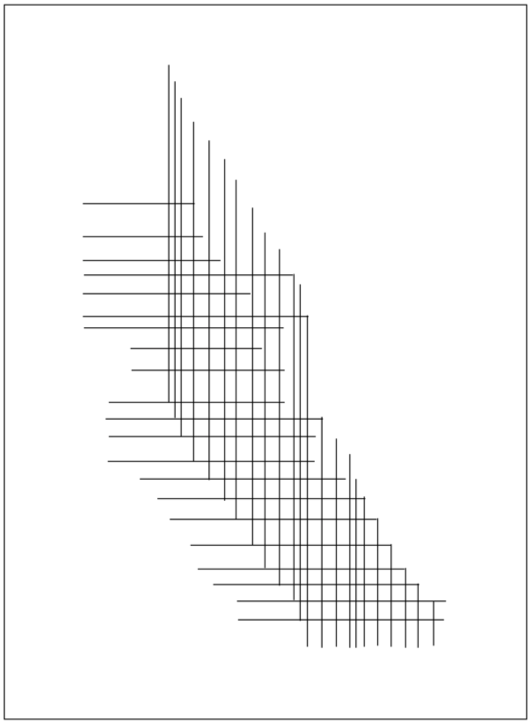

**FIGURE 4.** The tree of overtones in a simpler version. This figure illustrates a part of the pattern, as it would look on a millimeter paper.

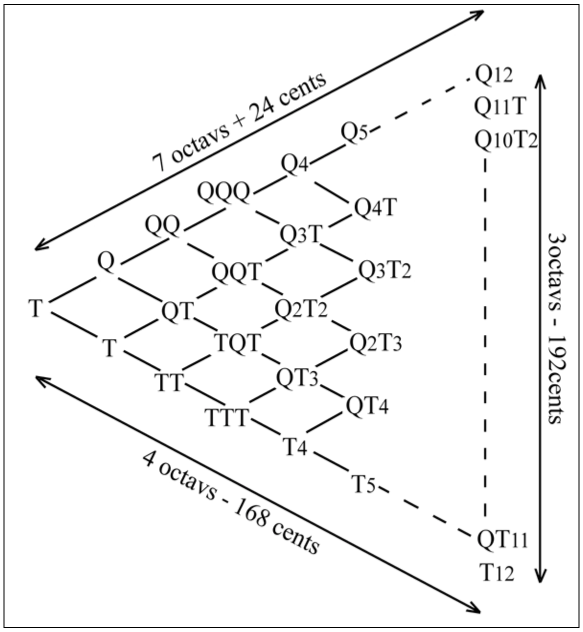

**FIGURE 5.** An equational representation of the truncated tree of overtones, including information about the deviations from the simple 12-tone system. The pattern is converted from a millimeter paper.

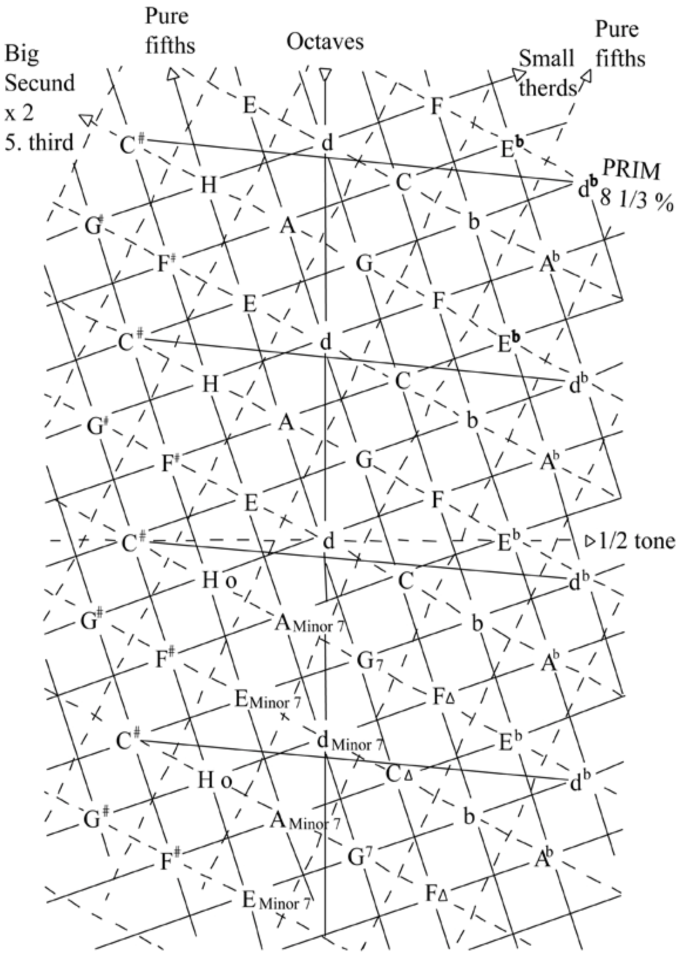

**FIGURE 6**. An illustration of the pattern of a classical tone tissue with all its intervals.

The idea is to release the whole language of musicality from the overtones, demanding that we discover the tones of the scale in a reasonable way. However, this has not yet been possible, indicating the exclusion of an important principle. What is found is all the triads and the natural ways to follow between these; namely, the above-described jumps of thirds and fifths. However, the scales and the complete melodic aspects are missing. Below, we will expand the system to include these concepts.

## The Reflection Around Tonic

In our system, we miss the foundation for minor, the fourth (F) and the small third. Leading the fifth downwards gives the fourth immediately, although this is not allowed according our rule that says that the overtones should be understood as being over the tonic. However, the brain could also have the capability of forming overtones downwards. So, we will try to make a functional reflection, making the downward fifth and third equal with the upward equivalents. Fig. 7 shows that this gives the tone F as the missing downwards fifth of the tonics (Cs). F (the subdominant) is an important tone, ranging exactly under the fifth. If the third, however, is reflected, the result will be the tone G, not necessary to form C major. The problematic tone G interferes with the idea that a simple reflection can eliminate our problems, that two huge thirds sound awful together. Expressed in another way, two huge thirds give a very tense sound only

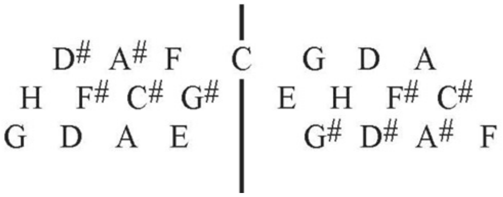

**FIGURE 7.** A reflection of the system around the tone C.

enjoyable when the tension brakes. The question, then, is which sound is the most beautiful? The simplest or the most complex, tensioned and disharmonic. We think the answer is that the disharmonic sound can be separated in harmony (discussed later).

## The Complex Overtone Multidimensionality

The simple reflection could not solve our problem and we still need to find a useable expansion to our system. An intimate investigation of the number of overtones shows that the first octave (sounds boring and normally overlooked) in the reality is stretched out over two complete tone tissues, analogous to those illustrated in Fig. 5 (see also Figs. 8A and B). Together, these two-tone tissues may cover more than the fifth-third tone tissue because the octave is more fundamental and of higher priority than the fifth and third. Furthermore, seventh (the sixth overtone) is stretched out entirely over three tone tissues, namely the tone tissues of the octave, the fifth, and the third (Figs. 8C–E). All the following overtones, consisting of a number of nodes equivalent to the prime numbers, similarly stretch out an expanded number of tone tissues. In this way, the theoretical infinite number of overtones stretches out as still a larger number of tone tissues, all being tied in a huge multidimensional structure sourcing all musical experiences. We call this the complex overtone multidimensionality.

Because all nodes represent a number of overtones, the complex overtone multidimensionality can be simplified as a roomy latticework. Professor Yngve Trade (personal communication) has explained to us that overtones are normally organized with seven or more nodes in the sound universe instead of in the harmonic world. Such reduction of the complex overtone multidimensionality makes it exactly three-dimensional and related to our well-known three-dimensional visual world. Therefore, the harmonic universe is exactly experienced as a roomy development contrary to the rhythm's development in time. This makes a 3-dimensional model of the reduced complex overtone multidimensionality in which the number of overtones are presented as nodes (see Fig. 9). In this model, the reduced complex overtone multidimensionality is expanded from a reflection of the fifth and third, also to include the octave and (in principle) all overtone intervals of the tissue. It should now be possible, in principle, to move musically in all directions from a selected tone. Practically, we prefer to move the tone height upwards from the musical starting point. Such action looks to be attached to the functional dynamics operating around the complex overtone multidimensionality that makes the music. By this, the reduced complex overtone multidimensionality makes only the frame around the music, while the dynamics and the fundamental functions make the content. In a philosophic way, the understanding of the complex overtone multidimensionality can be interpreted as the structure in the human brain that the emotions use to create the music.

The three-dimensional model of the sound-reducing complex overtone multidimensionality gives a complete new and clear understanding of the concept, to temperate the music, possibly. Already, the fifth-third tone tissue in Fig. 5 shows that it would be possible to temperate the system especially by stretching the fifths ('stretching' meaning making them artificially larger than they are in nature), 82 tones are reduced to only the 12 tones of the normal scale as the transformation makes most of the different tones identical, but this is no argument for postulating that the brain should prefer the equally floating temperate scale.

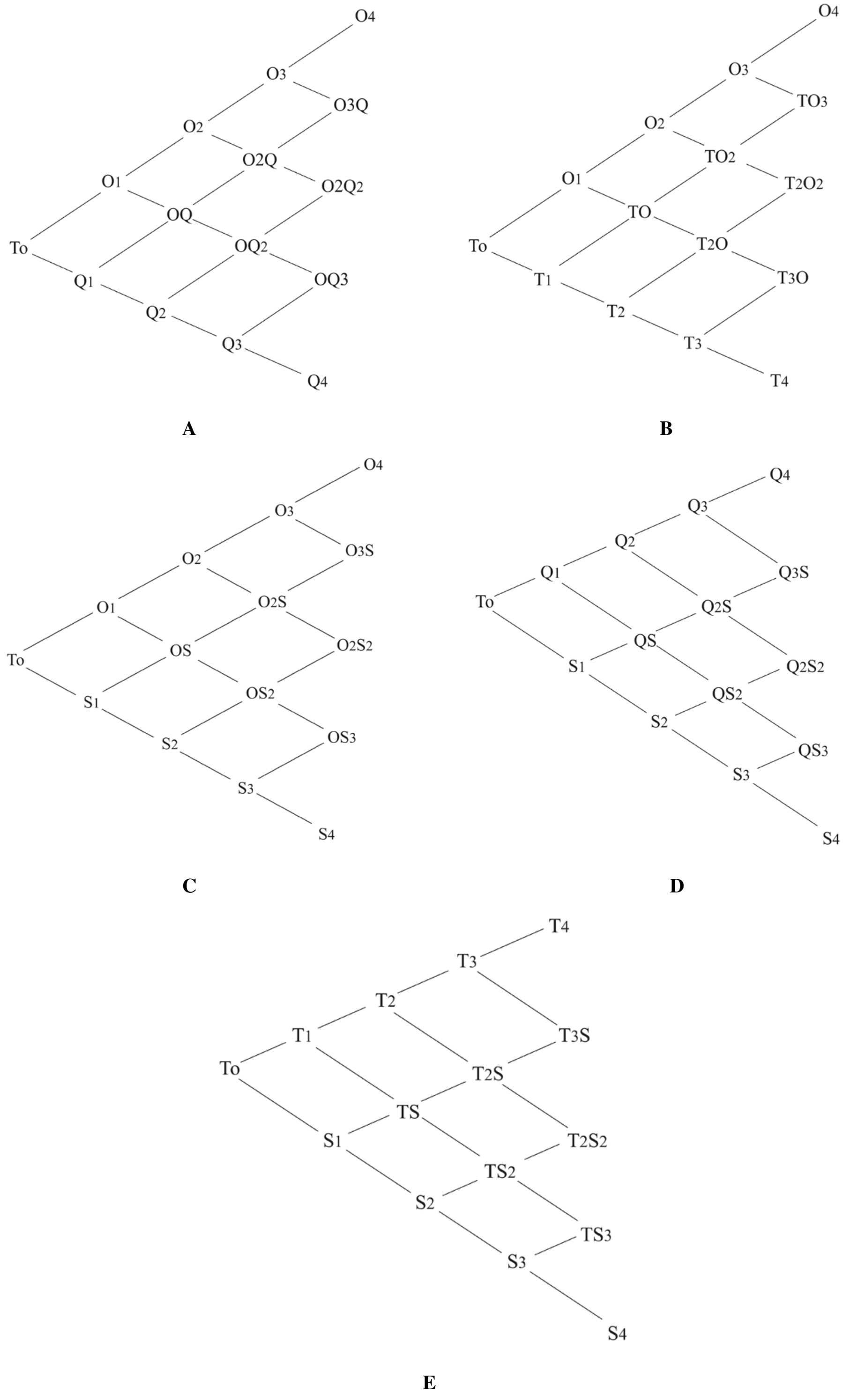

**FIGURE 8**. (A) The octave-fifth tone tissue. (B) The octave-third tone tissue. (C–E) The seventh’s tone tissues.

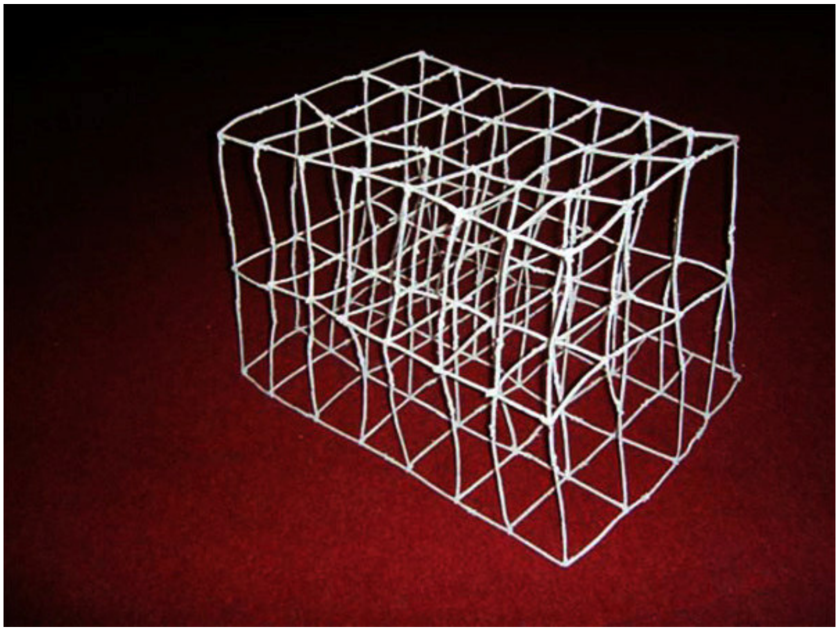

**FIGURE 9.** The reduced complex overtone multidimensionality as a three-dimensional roomy representation of the harmonic sound universe of a human being.

However, from the model, we can see that the number of octaves forms the most fundamental structure in the complex overtone multidimensionality. If we project the fifths and the thirds on this structure that is already contained by the complex overtone multidimensionality (this may be reasonable because it would simplify the system and make it harmonic), it will result in an equally floating temperate chromatic scale. This is illustrated by folding our complex overtone multidimensionality model (see Fig. 10). This interpretation is the condition of all scales and the scale is the condition for the melodic tone world[9]. By this, the melodic concept can be understood as a freedom within the borders of our musical tolerance, when we choose not to use the roomy expression of the harmonies. Through a melody, we can get a compact harmonic series of tones presented in a high speed. In this way, the harmonic series of tones are reduced to weak memories about harmony. Thereby, the human being gets the joy of being able to experience huge sequences of tones. Concerning the conditions between melody and harmony, this explains how the melody gets its huge degree of freedom compared to the harmony.

## The Release of the Scale of Major and Minor from the Model of the Complex Overtone Multidimensionality

The up-folding of the three-dimensional complex overtone multidimensionality around the octave axis gives a chromatic scale without any structures of major and minor. Therefore, this will not be able to source these tones. The major is, in a way, formed out of the fifth and third because it is defined by a huge third as a beginning point in the same way as the minor is defined with a little third as a beginning point[8,9]. The major and the huge third are involved in our terms, but the little third is not. The little third exists only as the difference between the fifth and the huge third. This difference indicates that one of the intervals should go up and the other down. This again indicates that going exactly a third up and a fifth down leads to the three parallel kinds of minor tones. If we only go one fifth down we will, together with the tonic (C), get the three kinds of major tones (see Fig. 11). It is exactly the mentioned jump that makes the “well-known” tones. Here, the structure of the complex overtone multidimensionality gives the possibility of a number of combinations of theoretically equal fifth-third intervals (see Fig. 12).

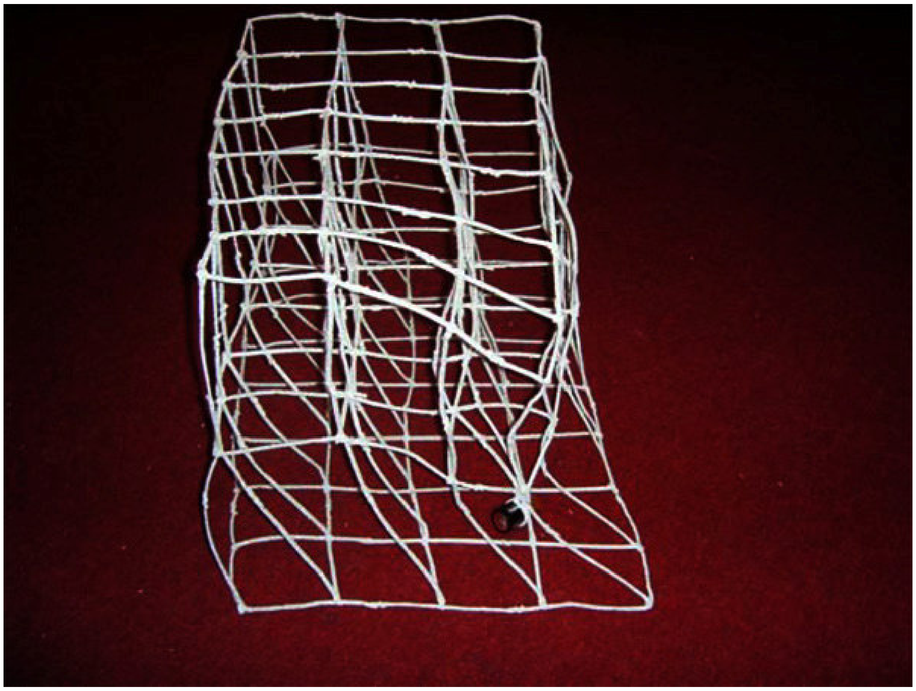

A

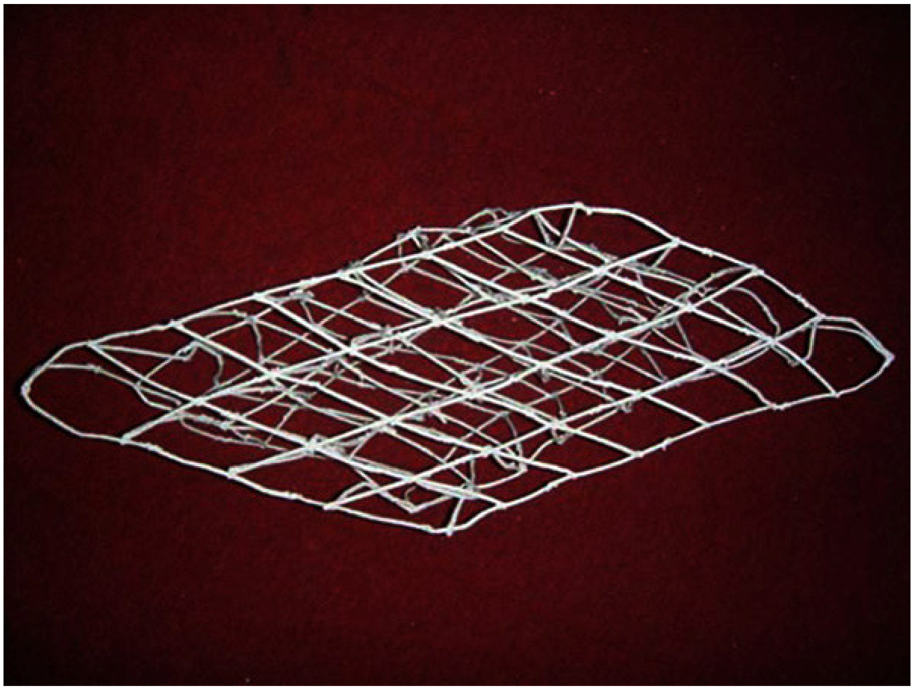

B

**FIGURE 10.** A folding of the complex overtone multidimensionality that projects all the tones on the octave axis and, by this, immediately forms an equally floating temperate scale. (A) A folding of a corner of the structure is transmitted throughout the structure. (B) A complete stretch of the system from the two horizontal corners.

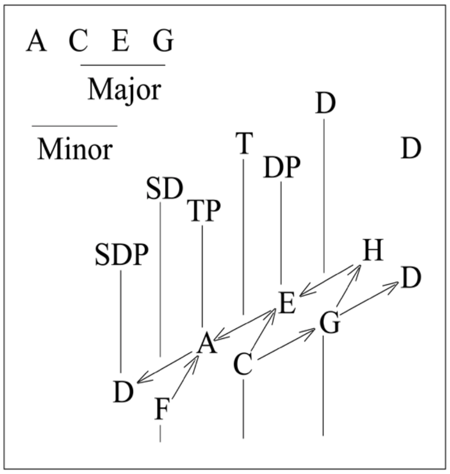

**FIGURE 11.** The strategy for the development of the major and minor parallelism.

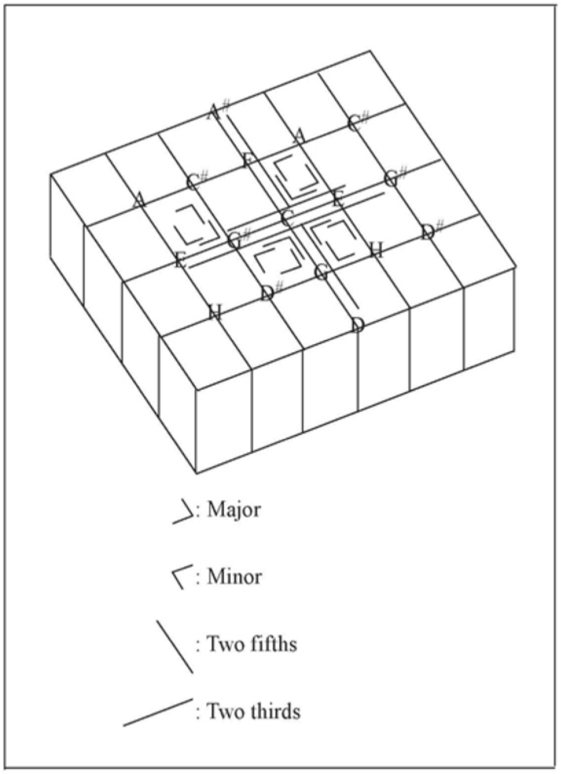

**FIGURE 12.** The theoretical possibilities for fifth-third interval jumps in the reduced complex overtone multidimensionality.

## Interval Complexity as Fundament of the Choices of Harmony Intervals

Are the intervals the final factor of the functional choice of intervals? This work has especially been based on computer analysis of the mutual conditions of the tones interference. This has led to the conclusion that, for isolated intervals, it counts that simplicity is beauty and complexity is ugliness. Furthermore, it is important to point out that this looks very different from a harmonic connection where the complexity is the same as musical tension, without which the music would be dull and boring. This shows that isolated intervals make the beautiful (resting) music, while the compact (tense) intervals make the ugly music.

## Interval Complexity as Fundament for the Harmonic Intervals

Our work (not published) concerning the conditions of interference in music has, as mentioned above, led to the conclusion that for isolated intervals, simplicity is beauty and complexity is ugliness. However, in a harmonic connection where the complexity is equivalent with musical tension, without which the music would be colorless and boring, these relations are somewhat reversed. The real beauty of the music seems to come from transformation of disharmony into harmony through time. When we forget the dimension of time and dynamic developments, the simple and harmonic becomes the beautiful. There seems to be an objective, static, beauty of simple intervals, but in addition, an objective dynamic beauty comes from the gradual approximation to more simple intervals through the time.

Below, we will try to show that the simple intervals are the beautiful ones. Simplicity results from a pattern of intervals that happen through addition of the difference in the sinus curves between two different tones, because these contain only a few simple vibrations in a pattern that repeats itself very quickly. Therefore, beauty is represented by a periodic function and the periods are only brief when an interval is harmonic.

## The Possibilities for Interval Composition in the New Model

Fig. 13 shows the triads that could form the tone C. Theoretically, the following are possibilities for combination of the fifth-third tone tissue: two fifths on line, two thirds on line, and the ones that form an angle, an upwards fifth and an upwards third, a downwards fifth and a downwards third, and the two combinations of an upwards and a downwards fifth and third.

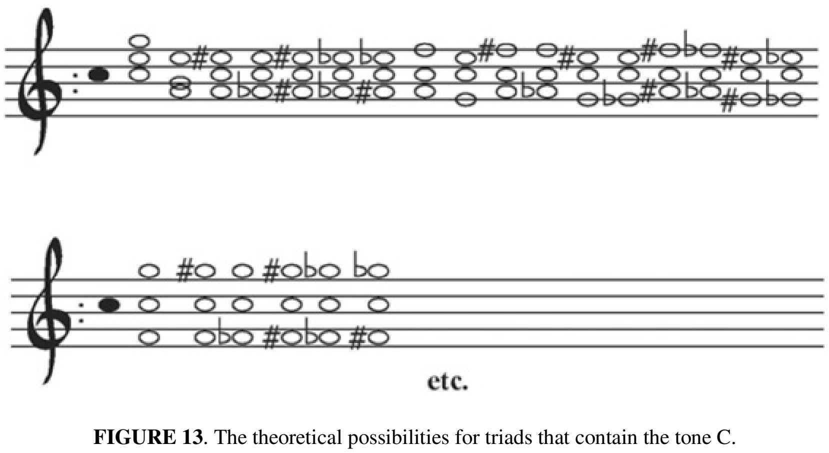

**FIGURE 13**. The theoretical possibilities for triads that contain the tone C.

First we analyze the two fifths and the two thirds. None of these intervals is experienced as beautiful, when standing alone. The difference between the two fifth intervals is called a “none” (nine tones), and the difference between the two third intervals is a fifth. The straight lines in Fig. 13, therefore, form a great amount of disharmony. When the third and the fifth of the third, or fifth and the third of the fifth, are gathered, the interval of half tones are also gathered. This interval looks to be very complex. A tension so huge demands a great musical talent to dissolve it. Therefore, this interval is very seldom used and, standing alone, it is one of the ugliest intervals.

A fifth and an upwards third form the well-known and harmonic triad, the fundament of the major. This can also be formed by the combination of a downwards fifth and an upwards third, but this combination has another keynote. The combination with the upwards fifth and the downwards third forms the small third, the fundament for the minor triads.

## DISCUSSION

Our ability to understand perception of music from simple mathematical analysis indicates that the human brain uses a simple mathematical language to interpret the world. We have not yet identified this language, but from what we learned in this paper, the brain seems to use a fractal model of reality, like a tree with many branches, but going not just up like a normal tree, but also going down from the point of focus. We have seen quantities turning into qualities, which seems to be a universal feature of how the nervous system creates meaning out of chaos in the world.

Our analysis of music seems to confirm our understanding of the brain as presented in an earlier paper[10]. Seemingly, the functioning brain behaves like one highly-structured, but extremely coherent, ball of energy, making the idea of a quantum field that carries the consciousness very likely[11,12,13,14,15,16,17,18,19,20,21]. If that is the case, then the structure of the experience of music might be a quite unique window into a hidden quantum reality of brain and life. The structure of music is then a mirror of a much more complex, but similar, structure of the energetic field of the working brain.

The minor and major intervals are the fundaments of the most beautiful scales of music. The minor intervals are more complex than the major intervals and it can be discussed if their well-known “saddening” sound is grounded in this complexity (a disharmony) or in the fact that they are constructed by a downwards third that could symbolize a downward aim, or movement towards the earth.

When we move upwards in the system of tones (common major), we feel energized and happy, and this upward move adds speed and movement in the music. The picture of a downward movement is often experienced as a loss of energy and a slowing down. If the minor was sad because it contains the small disharmony, maybe this could be dissolved to major by going from the small to the huge third. This, however, seldom happens.

If the minor is saddening because it represents an energetic downward movement, this demands a fundamental reinterpretation of how the brain creates music; we suggest the existence of a multidimensional perceptional space, very similar to the complex tree of overtones.

We know that our analysis of music is not yet thorough enough to answer the fundamental questions of music satisfactorily. We have found proportionality between the complexity of the intervals and their subjective tension and beauty. A harmonic tension (a musical ugliness), when isolated, could be experienced as a painfully bad sound, but could also be transformed in a piece of music into a wonderful, harmonic, dynamic, and moving energy.

We learn, yet again, that the interesting human experience is created at the border between chaos and order, and we always love when we go from chaos to order. However, without darkness and disharmony, life is just a dull and indifferent affair.

## ACKNOWLEDGMENTS

Professor Yngve Trade has been so kind to discuss the proposed model and its implications with us. The research was supported by grants from IMK Almene Fond. Our research on quality of life has been approved by the Copenhagen Scientific Ethical Committee under number (KF)V.100.2123/91.
